# Effects of Temperature and Wetness Duration on Infection by *Coniella diplodiella*, the Fungus Causing White Rot of Grape Berries

**DOI:** 10.3390/plants10081696

**Published:** 2021-08-18

**Authors:** Tao Ji, Luca Languasco, Ming Li, Vittorio Rossi

**Affiliations:** 1 Department of Sustainable Crop Production, Università Cattolica del Sacro Cuore, Via E. Parmense 84, 29122 Piacenza, Italy; tao.ji@unicatt.it (T.J.); luca.languasco@unicatt.it (L.L.); 2 National Engineering Research Center for Information Technology in Agriculture (NERCITA)/National Meteorological Service Center for Urban Agriculture, China Meteorological Administration & Ministry of Agriculture and Rural Affairs, Beijing 100097, China; lim@nercita.org.cn; 3 Collaborative Innovation Center for Green Prevention and Control of Forest and Fruit Diseases and Insect Pests, Beijing Academy of Agriculture and Forestry Sciences, Beijing 100097, China

**Keywords:** *Coniella diplodiella*, environmental factors, infection pathway, inoculum dose, incubation

## Abstract

Grapevine white rot, caused by *Coniella diplodiella*, can severely damage berries during ripening. The effects of temperature and wetness duration on the infection severity of *C. diplodiella* were investigated by artificially inoculating grape berries through via infection pathways (uninjured and injured berries, and through pedicels). The effect of temperature on incubation was also studied, as was that of inoculum dose. Injured berries were affected sooner than uninjured berries, even though 100% of the berries inoculated with *C. diplodiella* conidia became rotted whether injured or not; infection through pedicels was less severe. On injured berries, the disease increased as the inoculum dose increased. Irrespective of the infection pathway, 1 h of wetness was sufficient to cause infection at any temperature tested (10–35 °C); with the optimal temperature being 23.8 °C. The length of incubation was shorter for injured berries than for uninjured ones, and was shorter at 25–35 °C than at lower temperatures; the shortest incubation period was 14 h for injured berries at 30 °C. Mathematical equations were developed that fit the data, with R^2^ = 0.93 for infection through any infection pathway, and R^2^ = 0.98 for incubation on injured berries, which could be used to predict infection period and, therefore, to schedule fungicide applications.

## 1. Introduction

White rot of grapevines is also known as “hail disease” because it frequently develops following hailstorms [1]; the disease, however, can develop even in the absence of hailstorms [2]. White rot, which is caused by the fungus *Coniella diplodiella* (Speg.) Petrak and Sydow (syn. *Coniothyrium diplodiella* [Speg.] Sacc. and *Pilidiella diplodiella* [Speg.] Crous and van Niekerk; [3]), was first described in 1878 in Italy [4], and is currently distributed worldwide [5,6,7]. In addition to *C. diplodiella*, other species of *Coniella*—including *C. vitis* [6] and *Pilidiella castaneicola* [8]—can naturally infect vines in the vineyard, or can infect grapevines in inoculation studies in the case of *C. petrakii* and *C. fragariae* [9].

Grapevine white rot affects all green tissues of the vine, but mainly damages ripening clusters. Yellowish and water-soaked lesions appear on the edges or tips of the leaves, which gradually expand inward, forming concentric, wheel-shaped lesions that eventually dry; grayish-white pycnidia can appear on the diseased tissue in moist weather [10,11]. Long, depressed, brownish necrotic areas may also occur around the nodes of green shoots that can evolve in cankers [2]. As previously noted, however, the typical symptoms appear on clusters. The pathogen forms yellowish-brown and water-soaked areas that rapidly enlarge to cover the whole berry, which finally becomes soft and rotten. In a later stage, the berry’s surface is densely covered with small, brown-violet pustules consisting of immature pycnidia that rise from the epidermal layers of the cuticle without rupturing it; the layer of air between the cuticle and the epidermis plus the grayish-white color of the mature pycnidia make the infected berries appear white, which explains why the disease is named white rot [2,12].

*C. diplodiella* overwinters as mycelia or pycnidia in infected canes, rachises, and mummified berries that fall into the soil [13], where pycnidia can remain viable for more than 15 years, and can repeatedly discharge conidia under moist conditions [2,14]. Conidia (and soil particles containing conidia) are transported to plant surfaces by splashing rain or farm equipment. Conidia then germinate and cause infection through stomata [5,10], microcracks, or mechanical wounds [4,10], or via direct penetration [4].

White rot is often closely associated with hailstorms that occur during berry ripening, because hail causes wounds that facilitate the infection of berries. In areas where hailstorms are sufficiently frequent to cause a buildup of *C. diplodiella* inoculum in the vineyard, a single-spray application of a fungicide is recommended as soon as possible after the storm; the recommended fungicides include folpet, dichlofluanid, or thiram [15]; captan and dicarboximides [2]; chlorothalonil [13]; and tebuconazole, pyraclostrobin, carbendazol, and mancozeb [16]. In affected vineyards, however, summer rain followed by persistent moisture and temperatures of 24–27 °C can occasionally lead to disease outbreaks [2]. In some viticultural areas, the disease occurs almost every season. For instance, grape white rot has been reported as one of the main fungal diseases affecting grapes in China [11,17,18], where it causes an annual production loss of 16% [17]. In these cases, repeated fungicide applications are required to control the disease [19].

Knowledge of weather conditions leading to infection may be important for timing both single hail-related fungicide applications and repeated fungicide applications. Researchers have found that infection occurs over a wide temperature range, from <12 °C and >33 °C, with the optimum between 24 and 27 °C [2,13,20,21]. Infection also requires a minimum of 2 h of wetness [10].

Knowledge about the pathways and environmental conditions for infection by *C. diplodiella* is crucial for disease control, but the available information is fragmented, inconsistent, and based on only a few early studies. We therefore conducted a study with detached grapevine berries under controlled environmental conditions to determine (1) the temperature and moisture conditions required for different infection pathways, and their interactive effects on disease severity; (2) the effect of inoculum dose on berry infection; and (3) the effect of temperature on incubation length.

## 2. Results

### 2.1. Effect of Temperature and Wetness Duration on Berry Infection (Experiment 1)

**Figure 1:**
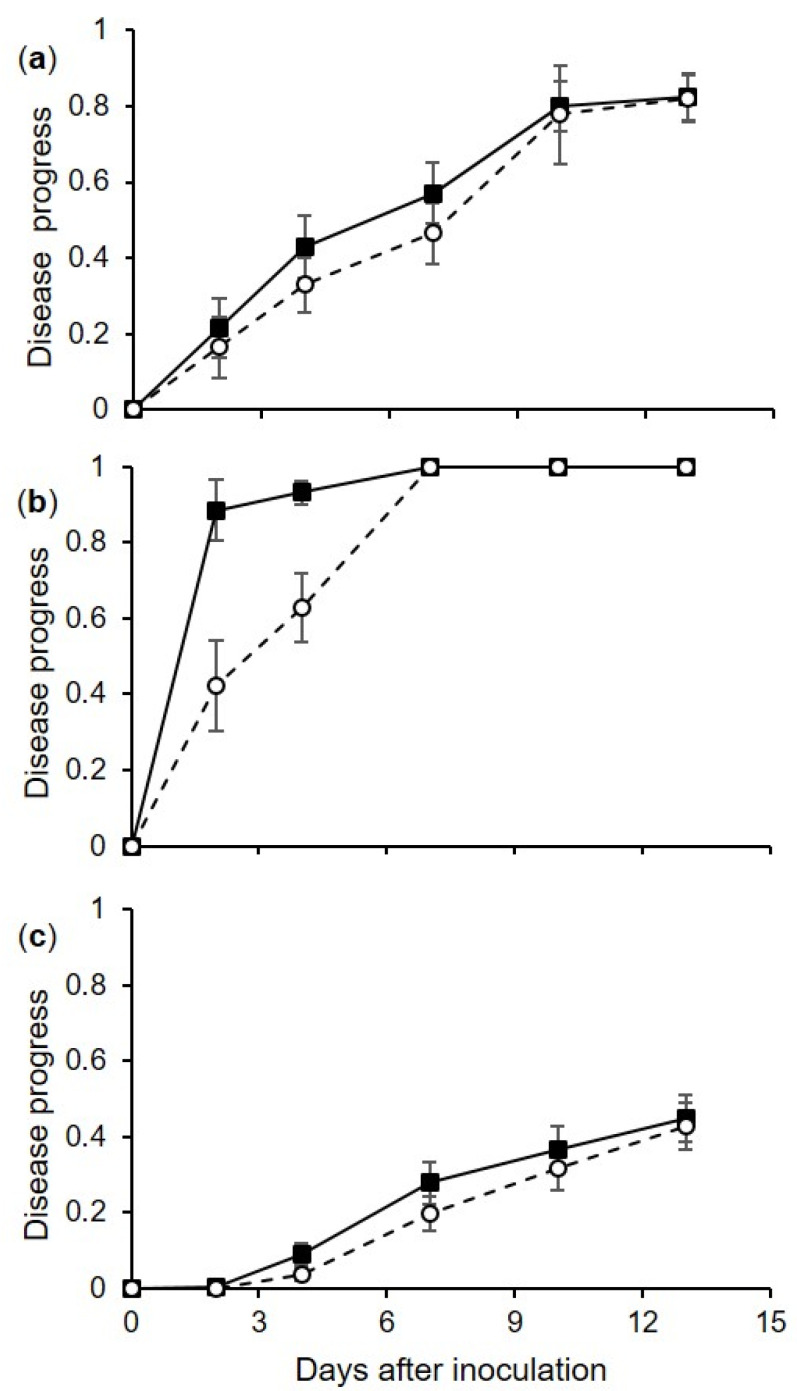
Progress of disease incidence (as the proportion of berries showing symptoms, on a 0–1 scale, where 1 means all of the berries are affected; black squares) and severity (as the proportion of the berry surface showing symptoms, on a 0–1 scale, where 1 means all of the berry surface is affected; white circles) on grapevine berries inoculated with conidia of *Coniella diplodiella* (experiment 1). After uninjured berries (**a**), injured berries (**b**), or pedicels of uninjured berries (**c**) were inoculated, the berries were kept at 30 combinations of 6 temperatures (T: 10, 15, 20, 25, 30, and 35 °C) and 5 wetness duration periods (WD; 1, 3, 7, 12, and 24 h); each combination was represented by 3 replicates, with 15 berries per replicate. In each panel, values are means (±SE, n = 90) of disease incidence or severity with the 6 temperatures and 5 wetness duration periods.

**Figure 2:**
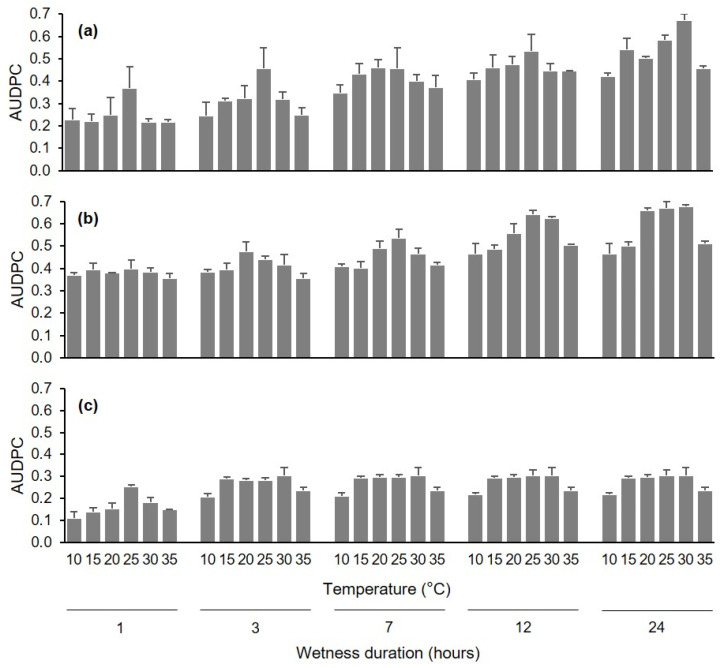
Values of the area under the disease progress curve (AUDPC, dimensionless) for grapevine berries inoculated with conidia of *Coniella diplodiella* (experiment 1). After uninjured berries (**a**), injured berries (**b**), or pedicels attached to uninjured berries (**c**) were inoculated, the berries were kept at 30 combinations of 6 temperatures and 5 wetness duration periods. Values are means (± SE, n = 45) of AUDPC data. AUDPC data were standardized by dividing the original AUDPC values by the time considered for their calculation, i.e., 10, 4, and 13 d for A, B, and C, respectively.

The disease progressed very rapidly when berries were injured before they were inoculated with *C. diplodiella* conidia; 90% of the berries had initial water-soaked areas at 1 d after inoculation, and all of the injured berries were completely rotten after 7 d (Figure 1b). Disease progress was slower for uninjured berries, with approximately 80% of berries showing symptoms on the majority of the berry surface after 10 d (Figure 1a). Disease progress was slowest for pedicel inoculation, with less than 50% of the berries affected after 13 d (Figure 1c). The AUDPC values were then calculated at 4 d for injured berries, 10 d for uninjured berries, and 13 d for pedicel-inoculated berries, and then standardized as previously described (Figure 2).

The ANOVA carried out on standardized AUDPC data showed that all of the main sources of variation affected disease progress with *p* < 0.001. Infection pathway accounted for >50% of the variance, with the AUDPC highest for injured berries (overall average 0.476 ± 0.011), intermediate for uninjured berries (0.395 ± 0.014), and lowest for pedicel inoculation (0.249 ± 0.007). Duration of the wet period (WD) accounted for 27.9% of the variance, and the pathway × WD interaction accounted for 5.8% of the variance. For any infection pathway, the AUDPC for berries that were kept wet for only 1 h after inoculation was approximately 50% of the AUDPC for berries that were kept wet for 24 h after inoculation (Figure 2). The AUDPC then increased with increasing WD up to 24 h, except that the AUDPC for pedicel inoculation did not increase further after a 3 h WD (Figure 2). Temperature during the wet period accounted for 7.9% of the variance, and the infection pathway × T interaction was not significant (*p* = 0.086). For all infection pathways, the optimal temperatures for infection were therefore between 20 and 30 °C; at 10 and 35 °C, the AUDPC values were significantly lower than at the optimal temperature range (*p* < 0.001). The infection pathway × WD × T interaction was significant at *p* = 0.044, but accounted for only 4.1% of the variance.

**Figure 3:**
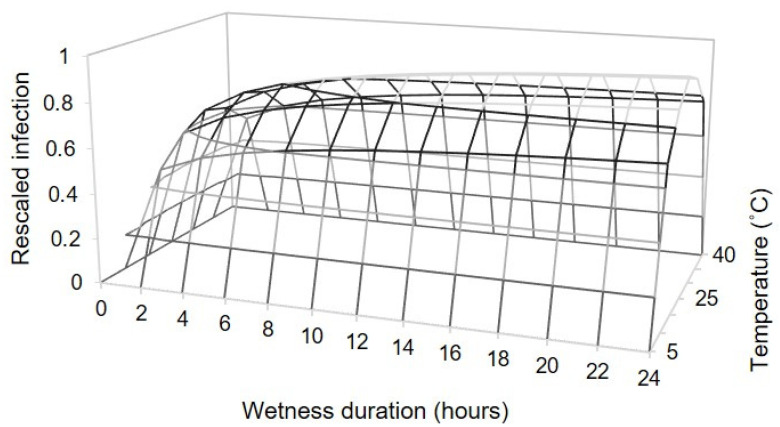
Effects of temperature and wetness duration on the rescaled infection severity in grapevine berries inoculated with conidia of *Coniella diplodiella*, as estimated by Equation (2) and with data from experiment 1. After uninjured berries, injured berries, or pedicels attached to uninjured berries were inoculated, the berries were kept at 5 wetness duration periods (1–24 h) and at 6 temperatures (10–35 °C). For fitting purposes, AUDPC data were rescaled on a 0–1 scale by dividing the original AUDPC values by the maximal value for each infection pathway (uninjured, injured, and pedicels).

The estimated parameters for Equation (2) were Tmin = 5 °C, Tmax = 40 °C, *a* = 5.067 ± 1.777, *b* = 1.162 ± 0.088, *c* = 0.350 ± 0.054, *m* = 0.468 ± 0.286, and *n* = 0.363 ± 0.092, which provided a three-dimensional representation of the changes in the relative infection severity based on the combined effect of temperature and wetness duration (Figure 3), with R^2^ = 0.93, CCC = 0.96, RMSE = 0.10, and CRM < 0.0001. The plot of predicted versus observed values did not show systematic deviations (not shown). For the whole dataset, the intercept of the regression line of predicted versus observed data was α < 0.0001 (not significantly different from α = 0, at *p* = 0.998) and slope β > 0.999 (not significantly different from β = 1, at *p* = 0.982).

Based on Equation (3), the optimal temperature was Topt = 23.8 °C, with a 95% confidence interval from 22.4 to 25.0 °C.

### 2.2. Effect of Inoculum Dose on Berry Infection (Experiment 2)

**Figure 4:**
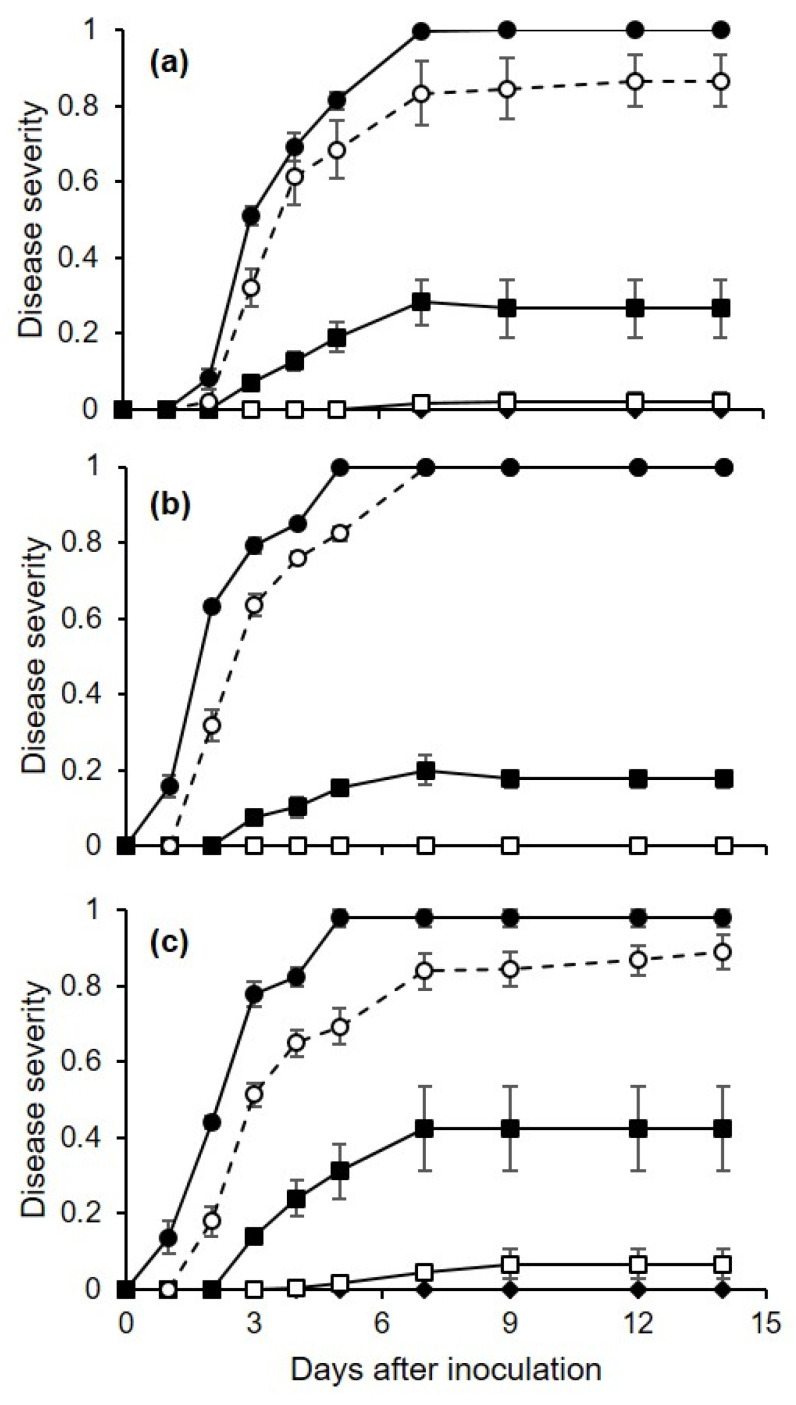
Progress of disease severity (as the proportion of berry surface showing symptoms, on a 0–1 scale, where 1 means that all of the berry surface is affected) on grapevine berries that were injured and then drop-inoculated with a suspension of *Coniella diplodiella* that contained 10 (black diamond), 10^2^ (white squares), 10^3^ (black squares), 10^4^ (white circles), or 10^5^ (black circles) conidia/mL (experiment 2). After inoculation, berries were incubated for a 24 h wet period at 15 (**a**), 25 (**b**), or 35 °C (**c**). Values are the means (± SE, n = 45).

The progress of disease severity on the berries that were injured and then drop-inoculated with conidia of *C. diplodiella* differed depending on the inoculum dose and the temperature at which the berries were incubated following inoculation (Figure 4). Specifically, disease severity increased very rapidly in the first 7 d following inoculation when the inoculum dose was high (10^4^ and 10^5^ conidia/mL), and with temperatures of 25 and 35 °C; in these treatments, the disease did not substantially increase in the next 7 d (Figure 4). No or very light disease was observed at low inoculum doses (10 or 10^2^ conidia/mL, respectively), regardless of temperature (Figure 4).

**Figure 5:**
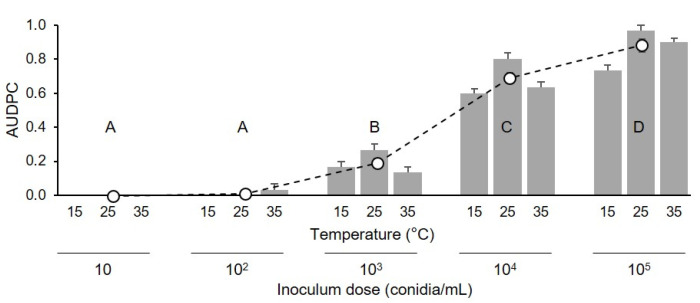
Values of the area under the disease progress curve (AUDPC, dimensionless) for grapevine berries that were injured and then drop-inoculated with conidia of *Coniella diplodiella* at 5 inoculum doses (10, 10^2^, 10^3^, 10^4^, or 10^5^ conidia/mL), and then incubated at 3 temperatures (T: 15, 25, or 35 °C) for 24 h (experiment 2). AUDPC data were standardized by dividing the original AUDPC values by the time considered for their calculation, i.e., 9 d. Bars indicate means (± SE) of 3 replicates, with 15 berries per replicate. White dots are the means (± SE, n = 45) of the 3 incubation temperatures for each inoculum dose; letters show comparisons of the latter means as determined by Tukey’s HSD test, with *p* = 0.05.

The ANOVA of the normalized AUDPC data showed that the disease was significantly (*p* < 0.001) influenced by inoculum dose—which accounted for approximately 97% of the variance—and by temperature, but not by their interaction. Based on the ANOVA, the AUDPC decreased as the inoculum dose decreased (Figure 5), and the disease was more severe at 25 or 35 °C (average of normalized AUDPC = 0.38 ± 0.11 and 0.37 ± 0.09, respectively) than at 15 °C (0.30 ± 0.08).

### 2.3. Effect of Temperature on Incubation Length (Experiment 3)

**Figure 6:**
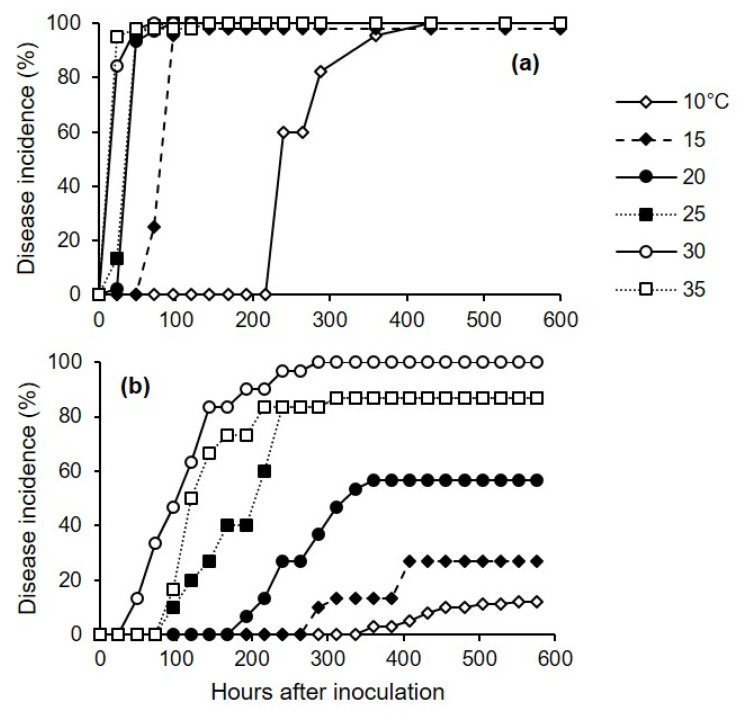
Percentage of injured (**a**) and uninjured (**b**) grape berries showing white rot symptoms following artificial infection with *Coniella diplodiella* conidia (experiment 3). After they were inoculated, berries were initially kept at 30 °C and with 100% relative humidity for 12 h to facilitate infection, and were then kept at one of the six indicated temperatures.

Lesions on injured berries were first observed at 24 h after inoculation with *C. diplodiella* when the injured berries were kept at 20–35 °C (Figure 6a). The water-soaked lesions first appeared near the wound, and gradually extended to cover the entire berry surface, and the berries subsequently became soft and rotten (yellow to pink) in the next 1–2 weeks. On uninjured berries, symptoms first appeared after 48 h at 30 °C (Figure 6b). Disease symptoms developed more rapidly on injured than on uninjured berries (Figure 6a,b). For both injured and uninjured berries, the disease developed faster at 25–35 °C than at 10–20 °C.

**Figure 7:**
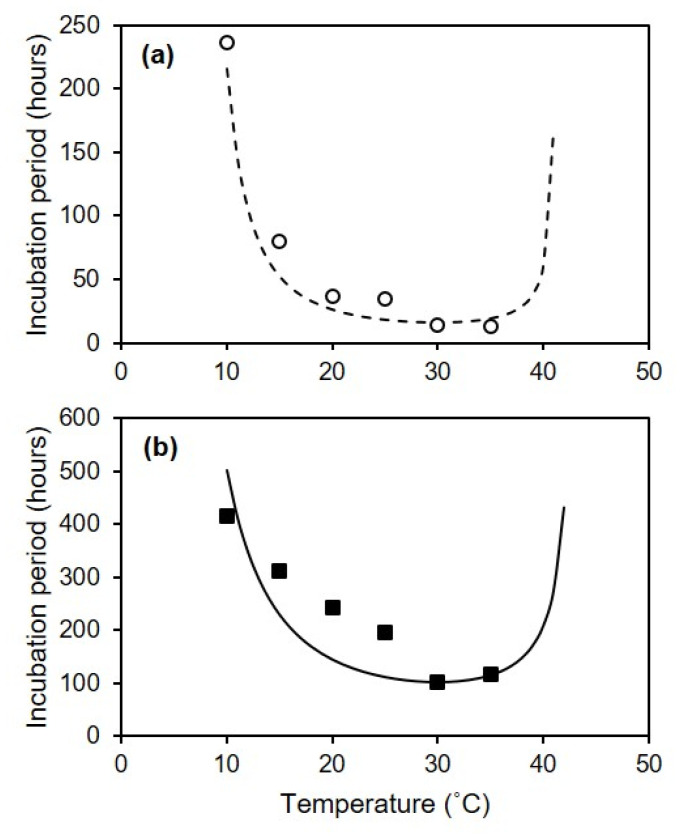
Effect of temperature on the length of the incubation period in grape berries that were injured (**a**) or uninjured (**b**) and artificially inoculated with *Coniella diplodiella* conidia (experiment 3). After they were inoculated, the berries were kept at 30 °C and with 100% relative humidity for 12 h to facilitate infection, and were subsequently kept at one of the six indicated temperatures. Period lengths were expressed as the number of hours required to reach 50% disease incidence (IP_50_). The lines show the fit of the data with Equation (5).

The incubation period was longest at 10 °C, with IP_50_ = 236 h for injured berries and 416 h for uninjured berries. The length of the incubation period decreased as temperature increased; at 30 °C, IP_50_ = 14 h for injured berries and 101 h for uninjured berries (Figure 7). The following parameter estimates of Equation (5) fit the incubation data for injured clusters with R^2^ = 0.98 and CCC= 0.98: IP_50_min = 16 h, Tmin = 5.5 °C, Topt = 30 °C, and Tmax = 41.5 °C (Figure 7a). Estimates for uninjured clusters (with R^2^ = 0.80 and CCC = 0.85) were IP_50_min = 101 h, Tmin = 1 °C, Topt = 30 °C, and Tmax = 43.5 °C (Figure 7b).

## 3. Discussion

In this study, we inoculated injured grape berries, uninjured grape berries, or berry pedicels with *C. diplodiella* conidia. To our knowledge, this is the first time different methods of *C. diplodiella* inoculation have been compared and the subsequent disease progress observed. In previous studies, inoculations were performed with mechanically wounded and chemically dewaxed berries [4], or with damaged berries only [5]. The information obtained by comparing different inoculation methods that represent the possible infection pathways in the vineyard increases our understanding of white rot’s epidemiology.

Berries that were injured before artificial inoculation with conidia of *C. diplodiella* were rapidly and severely affected, with some hyphae being visible on the inoculation site as early as 1 d after inoculation. The germination of *C. diplodiella* conidia, for example, was previously found to require external nutrients [14,22]. An early study found that germination was much more rapid in grape juice than in water, and that the minimal sugar concentration required for germination was 0.01% [5]. In another study, the germination rate increased linearly with sugar concentration [10]. Therefore, the presence of wounds on our mature berries likely provided nutrients that supported rapid germination of conidia, hyphal growth, and berry rotting. Our results are consistent with previous field reports that linked white rot outbreaks to hailstorms that occurred during berry ripening [1,2,5,11,23].

Uninjured berries were also severely affected in our experiments, but the disease developed more slowly on uninjured berries than on injured berries. Infection of uninjured berries may have occurred through microscopic cracks (“microcracks”) [10] that were not visible at the time of berry sampling in the vineyard. Microcracks in the cuticular membrane of berries increase susceptibility to pathogens [24]. Microcracks occur naturally on ripening berries, but their causes are not well understood [25,26]. Because *C. diplodiella* requires a high sugar concentration for conidial germination [10], and does not grow on the berry surface [4], penetration through microcracks may occur for those conidia that deposit near microcracks. Under natural conditions, the probability that conidia are deposited near microcracks may be much lower than in the current study, because we sprayed the entire berry surface with *C. diplodiella* conidia, and this may have resulted in a higher probability that some conidia were located near microcracks. Under natural conditions, the probability that conidia deposit near to microcracks may depend on both the number of conidia depositing on the berry surface and the frequency of microcracks. Becker and Knoche [25] found that the number of microcracks per mm^2^ on “Riesling” grape berries ranged from 0.02 to 1.35, and that the value depended on the orientation of the berry in the cluster and berry region (stylar scar, cheek, or pedicel end). The hypothesis that the probability of conidia landing near microcracks and infecting uninjured berries is low under natural conditions is supported by the steep decline in infection as the concentration of *C. diplodiella* conidia declined (Figure 4 and Figure 5).

Berries inoculated via the pedicel were also infected, but much less rapidly and less severely than injured or uninjured berries that were directly sprayed with conidia. The pedicel is a site where nutrients accumulate, because the retention of water on the pedicels may lead to the leakage of nutrients from the inner tissue, which may favor conidial germination and infection [10]. The development of water-soaked lesions in our berries, in fact, began at the base of the pedicels and gradually extended into the entire berry. That the fungus can infect through the pedicel and also through rachises was also observed by Locci and Quaroni [4].

In addition to infection pathways, our research provided quantitative data on the effects of temperature and moisture on berry infection. Irrespective of the infection pathway, *C. diplodiella* was able to cause infection under a wide range of temperatures, from 10 to 35 °C, with an estimated optimum of 23.8 °C, with a 95% confidence interval from 22.4 to 25.0 °C. The time required for the first visible symptoms to appear on artificially inoculated berries was dependent on temperature after infection, and was 1–7 d for injured berries and 2–14 d for uninjured berries; the overall time required was shorter at 20–35 °C and longer at 15 and 10 °C; this result clarifies the effect of temperature on infection, and also helps explain inconsistencies in previously published books and compendia [2,20,21]. For instance, Bisiach [2] indicated that the optimal temperature ranged from 24 to 27 °C, that the disease was slight above 34 °C, and that when the temperature fell below 15 °C for 24–48 h following a hailstorm, infection was negligible. In the field, the incubation period was reported to be 3–12 d, with the shortest durations at 25–27 °C [1,2,20,22]. We also found that a 1 h wet period was sufficient for infection via any of the three infection pathways and at any of the temperatures tested, and that the disease progression slightly increased with a longer wet period. To our knowledge, these are the first experimental data on the moisture requirements for *C. diplodiella* infection. The only previous data we found were from Chen et al. [10], who observed that 2 h of wetness following wounding was sufficient for a 59% disease incidence under field conditions.

Our overall results demonstrated that the infection of ripe grape berries can occur over a wide range of temperatures, and requires only a very short wet period. Considering that both hyphal growth and sporulation also occur between 10 and 35 °C [27,28,29,30]—i.e., at temperatures that are typical during berry ripening in the areas where grapes are grown [31,32]—and considering that short wet periods are likely to occur when splashes from rain disperse the conidia from the soil to clusters, we infer that the *C. diplodiella* infection cycle is not substantially limited by environmental conditions.

This provides insight into disease control. In locations where white rot outbreaks commonly follow hailstorms, growers usually apply fungicides after hail, but with inconsistent results. For instance, the efficacy was approximately 75% when folpet or captan was applied within 12–18 h after hail, but was only 0–50% when the treatments were applied 21–24 h after hail [33]. Dicarboximides were also effective when applied within 12–18 h after a hailstorm if temperatures were below 20 °C [2]. In early studies, interventions within 2–20 h after hailstorm [23,33,34,35,36] were often effective, those applied as late as 21 h after a hailstorm provided adequate protection, and those applied 24 h after a hailstorm were ineffective [37]. In other studies, however, treatments after 18 h were ineffective [33]. Our results indicate that fungicides should be applied as early as possible to control white rot following a hailstorm.

For the infections that are not related to hailstorms, and occur through pedicels and through microcracks on berry surfaces, disease control should be preventative, and should be based on one or more of the following: (1) reduction of inoculum; (2) prevention of microcracks on berry surfaces; and (3) fungicide application. The sources of primary inoculum of *C. diplodiella* are diseased rachises and mummified berries that have remained in the soil from previous seasons [2,22,38,39]; such inocula can remain viable for many years [13]. Inoculum can be reduced by the timely removal of affected clusters from the vineyard [22], or by soil disinfestation [38]. Microcracks on berry surfaces can be prevented by the careful management of water and fertilizer, the monitoring of soil moisture, and the proper spraying of growth hormones and micronutrients [10,26].

The equations we developed in this work provide an estimation of the combined effect of temperature and wetness duration on infection severity, and of temperature on the length of incubation. To our knowledge, this is the first time such equations have been developed. These equations could be used as risk algorithms [40] to predict infection period and to schedule fungicides accordingly. Further studies in vineyards are needed, however, in order to verify the utility of the equations.

## 4. Materials and Method

### 4.1. Fungal Isolates and Grapevine Cultivars

Three strains of *Coniella diplodiella* were used in this study: strain CBS166.84 was provided by Westerdijk Fungal Biodiversity Institute, and had been isolated from *Vitis vinifera* L. in Germany; strains COD2D and COD5A were isolated for the purpose of this study from grapevine cv. Merlot (*Vitis vinifera* L.), in Castell’Arquato in northern Italy. The identification of these strains was confirmed at the molecular level (see Supplementary Materials). Fungal colonies were maintained on water agar in tubes that were kept at 4 °C until used.

For the preparation of inoculum for the artificial inoculation of grape berries, 36-day-old cultures of the three fungal strains that had been grown on PDA at 20 °C were washed three times with 5 mL of sterile water and gently shaken. The resulting suspensions were passed through a double layer of sterile cheesecloth with sterile water. The suspensions of conidia were then adjusted to equal concentrations and were used in infection studies by mixing equal amounts of the three strains.

Two grapevine cultivars—Chardonnay and Ortrugo (*V. vinifera* L.)—were used; the vines had been planted in 2018 in a vineyard at Castell’Arquato, northern Italy (latitude 40°51′29″ N; longitude 9°51′17″ E, 258 m ASL). The vines were trained using a Guyot system (2.4 m between rows, 0.8 m between vines in rows) and were managed following the local practices, with soil management under the rows and natural grass growing in the inter-row space; no fungicides were applied starting from 45 d before the beginning of the study period. Thirty random clusters were sampled at ripening. For ”Chardonnay”, sugars were 18.1° Brix, pH was 3.2, and total acidity (as tartaric acid) was 6.4 g/L; for ”Ortrugo”, which is a local variety, sugars were 18.4° Brix, pH was 3.0, and total acidity was 6.0 g/L. The berries used for artificial inoculations were cut from clusters at their pedicels using scissors; all of the berries were turgid and visually disease-free with intact skins. In preliminary tests, the two cultivars showed high and similar susceptibility to *C. diplodiella*.

### 4.2. Effects of Temperature and Wetness Duration on Berry Infection (Experiment 1)

The first experiment assessed the effects of temperature (10, 15, 20, 25, 30, or 35 °C) and wetness duration (1, 3, 7, 12, or 24 h) on the infection of berries by *C. diplodiella*. Each of the 30 combinations of temperature × wetness duration was represented by 3 replicates, with 15 berries per replicate.

Berries with no visible damage and with their pedicels attached were washed in running tap water for 10 min, immersed in 75% ethanol for 1 min and then in 10% sodium hypochlorite for 1 min, and finally washed three times with distilled water. Berries were arranged over a metal mesh so that they did not touch one another, and the metal mesh was placed in an aluminum foil box with a wet paper towel on the bottom; a box was considered a replicate, and there were 15 berries per box. Berries were then inoculated as described in the next paragraph. After inoculation, boxes were sealed in a plastic bag to create a saturated atmosphere, and were kept in growth chambers at each of the 6 temperatures, under fluorescent light (12 h photoperiod).

At the end of each of the 5 wetness duration periods, berries were taken from the box and deposited on filter paper under a laminar air flow for 10 min to dry the berry surface; for injured berries (see below), the drop that exuded from the wound was dried by touching it with a piece of filter paper. The dried berries were then returned to the boxes, which were sealed and incubated at room temperature (25 ± 2 °C) with a 12 h photoperiod.

Berries were individually observed at 1–3-day intervals for 2 weeks to assess disease incidence and disease severity. Disease incidence was calculated as the percentage of berries showing yellowish-brown and water-soaked areas, and/or tissue softening and rot. Disease severity was assessed on individual berries as the percentage of the berry’s surface area showing disease symptoms by using a standard diagram that included the following classes: 0 (disease-free), 5, 10, 20, 30, 50, 75, and 100% (totally rotten). Disease incidence and severity were averaged for each replicate box.

We conducted experiment 1 three times, and we used three methods to inoculate the berries; these methods corresponded to the main infection pathways of *C. diplodiella*: (1) infection of uninjured berries; (2) infection of pedicels still attached to berries; and (3) infection of injured berries. For inoculation of uninjured berries (cv. Ortrugo), a conidial suspension (3 × 10^5^ conidia/mL) was uniformly distributed on berry surfaces with a hand sprayer (500 μL of suspension per box). For pedicel inoculation (cv. Ortrugo), a 20 μL drop of conidial suspension (3 × 10^5^ conidia/mL) was placed on the pedicel of each berry. For inoculation of injured berries (cv. Chardonnay), a sterilized needle was inserted through the skin and into the pulp of the berry, resulting in one wound per berry; a sterile pipette was then used to deposit a 20 μL drop of a conidial suspension (3 × 10^5^ conidia/mL) on the wound.

### 4.3. Effect of Inoculum Dose on Berry Infection (Experiment 2)

The second experiment assessed the effects of 5 inoculum concentrations (10, 10^2^, 10^3^, 10^4^, and 10^5^ conidia/mL) on injured berries. Injured berries (cv. Chardonnay) were inoculated as described previously (experiment 1), and were kept at 15, 25, or 35 °C for 24 h; afterwards, the drop that exuded from the wound was dried by touching it with a piece of filter paper. The dried berries were then returned to the boxes, which were sealed and incubated at room temperature (25 ± 2 °C) under fluorescent light (12 h photoperiod). The berries were then assessed each day for 15 days for disease incidence and severity, as described in the previous section. There were 15 combinations of inoculum concentration × temperature, and each combination was represented by 3 replicate boxes, with 15 berries per box. The experiment was repeated once.

### 4.4. Effect of Temperature on Incubation Period (Experiment 3)

Experiment 3 assessed the effect of temperature on the incubation period for injured or uninjured berries that were artificially inoculated with *C. diplodiella*. Berries were inoculated as described for experiment 1.

Experiment 3 was conducted twice, and we used two methods to inoculate the berries: In the first method, the conidial suspension was uniformly distributed on the surfaces of uninjured berries (cv. Chardonnay) using a hand sprayer (500 μL of suspension per box). In the second method, a sterilized needle was inserted into the pulp of the berry (one wound per berry) (cv. Ortrugo), and a 20 μL drop of the conidial suspension was deposited on the wound using a sterile pipette. After uninjured and injured berries were inoculated, boxes were sealed in a plastic bag to create a saturated atmosphere, and were incubated in growth chambers at 30 °C for 12 h to facilitate infection. The berries were then removed from the box and placed on filter paper under a laminar air flow for 10 min to dry the berries’ surfaces. The berries were then returned to the boxes, which were sealed and incubated in growth chambers at 6 temperatures (10, 15, 20, 25, 30, and 35 °C) under fluorescent light (12 h photoperiod); there were 3 replicate boxes (with 15 berries per box, and with uninjured and injured berries in separate boxes) for each combination of inoculation method and temperature treatment.

To assess the effects of inoculation method and temperature on the length of incubation, both injured and uninjured berries were individually observed daily for 24 d to assess disease incidence as the percentage of berries showing symptoms (as yellowish-brown and water-soaked areas, tissue softening, and rot).

### 4.5. Data Analysis

Disease severity data assessed at different times following inoculation of uninjured berries, injured berries, and pedicels under different conditions of wetness duration (WD) and temperature (T) were used to calculate the area under the disease progress curve (AUDPC) [41] by using the trapezoidal integration as follows:

AUDPC = ∑(y_i_ + y_i+1_)/2 × (t_i+1_ − t_i_) (1)

where y_i_ and y_i+1_ are the disease severity data (on a 0–1 scale) at two consecutive assessment times—i and i + 1, respectively [42]. Based on a preliminary data analysis of the disease curves (see Figure 1 and Figure 4), the AUDPC in the first experiment was calculated based on data collected at 10, 4, and 13 d after inoculation of uninjured berries, injured berries, and pedicels, respectively, and was calculated based on data collected at 9 d after inoculation in the second experiment. The AUDPC data were then normalized by dividing them by the time considered for their calculation [43].

Normalized AUDPC data were subjected to a factorial analysis of variance (ANOVA) to test the effect of infection pathway, WD, T, and their interactions in the first experiment, and the effect of inoculum dose, T, and their interaction in the second experiment. The ANOVAs were carried out using the SPSS package v. 19 (SPSS Inc., Chicago, IL, USA).

To study the relationship between temperature, wetness duration, and infection severity, we calculated a rescaled infection severity (0–1) by dividing the original AUDPC values by the maximal value in each replicated experiment (experiment 1). These data were then fitted by using following equation:

Y = (*a* Teq*^b^* (1 − Teq))*^c^* (1 − exp(−((*m* WD)*^n^*))) (2)

where Y is the rescaled infection severity; Teq is an equivalent of temperature, calculated as Teq = (T − Tmin)/(Tmax − Tmin), in which T is the mean temperature during the wet period (°C), and Tmin and Tmax are the minimum and maximum temperature, respectively; WD is the duration of the wet period (hours); and *a*, *b*, *c*, *m*, and *n* are the equation parameters. The parameter *a* is a proportionality factor, such that infection at the optimal temperature = 1; *b* is the parameter that regulates the infection severity increase between the minimal and the optimal temperature; *c* is the parameter that regulates the decrease from the optimal to the maximal temperature; *m* refers to the intrinsic rate of increase of the infection severity with respect to WD; and *n* is the growth rate. Tmin and Tmax were also estimated as model parameters.

The optimal temperature (Topt) was estimated as described by Analytis [44], based on the estimated values of *b*, Tmin, and Tmax in Equation (2), as follows:

Topt = *b*/(*b* + 1) (Tmax − Tmin) + Tmin (3)

The parameters of Equation (2) were estimated using the non-linear regression procedure of Origin 8 Pro (OriginLab Corporation, MicroCal). Goodness-of-fit of equations (e.g., Equation (2)) were determined based on the adjusted R^2^, the magnitude of the standard errors of the parameters, the root-mean-square error (RMSE), the coefficient of residual mass (CRM), and the concordance correlation coefficient (CCC) [45,46]. RMSE is the measure of the average distance between the real data and the fitted line [46]. CRM represents the tendency of the model toward over- or underestimation; more specifically, a negative CRM indicates that the model overestimates, while a positive CRM indicates that the model underestimates [46]. CCC estimates the difference between the fitted line and the perfect agreement line; a CCC value of 1 indicates that the fitted line is identical to the perfect agreement line [40,45]. The predicted values were then regressed against the observed values, and the null hypotheses that the intercept of the regression line was α = 0 and that the slope was β = 1 were tested using a *t*-test; when this test was not significant, both null hypotheses were accepted, and the model was considered a statistically accurate predictor of the real data [47].

The length of the incubation was expressed as the number of hours needed to attain 50% disease incidence by day 24 (hereafter referred to as the IP_50_). IP_50_ was calculated as follows:

IP_50_ = (d(x) × 24) + ((Y_d(24)_/2) − Y_d(x)_)/((Y_d(x+1)_ − Y_d(x)_)/24) (4)

where Y_d(24)_ is the final disease incidence (at day 24); Y_d(x)_ is the disease incidence at d x, i.e., the last d in which Y_d(x)_ < Y_d(24)_/2; and Y_d(x+1)_ is disease incidence at d x + 1, i.e., the first d in which Y_d(x+1)_ ≥ Y_d(24)_/2. For instance, if Y_d(24)_ = 100%, Y_d(9)_ = 0%, and Y_d(10)_ = 60%, then IP_50_ = (9 × 24) + ((100/2) − 0)/((60 − 0)/24) = 236 h.

The relationship between temperature and incubation length was fitted by using the equation of Magarey et al. [48] in the following form:

IP_50_ = *f*(T)/IP_50_min (5)

*f*(T)=((Tmax − T)/(Tmax − Topt))((T − Tmin)/(Topt − Tmin))^(Topt-Tmin)/(Tmax-Topt)^ (6)

where IP_50_min is the shortest duration of incubation; and Tmin, Topt, and Tmax are the minimal, optimal, and maximal temperatures, respectively. When T < Tmin or T > Tmax, *f*(T) = 0. IP_50_min and cardinal temperatures were estimated by evaluating the goodness-of-fit of a set of equations calculated by using an iterative procedure in which IP_50_min changed in 1 h steps and the cardinal temperatures changed in 0.5 °C steps.

## Data Availability

Data is contained within the article and Supplementary Material.

## Supplementary Materials

The following are available online at https://www.mdpi.com/article/10.3390/plants10081696/s1: Identification of *Coniella diplodiella* isolates.
