# Supplementary material for: Effects of Temperature and Wetness Duration on Infection by Coniella diplodiella, the Fungus Causing White Rot of Grape Berries
Source: Plants (Basel). 2021 Aug 18;10(8):1696. doi: 10.3390/plants10081696 (PMC8399050; doi:10.3390/plants10081696)
Supplement: Supplementary file 1 [file plants-10-01696-s001.zip › plants-1330691-supplementary.pdf]

Supplementary material

Identification of Coniella diplodiella isolates.

Strains COD2D and COD5A were isolated from grapevine cv. Merlot berries that showed typical white rot symptoms in August 2019, in a 15-year old vineyard located at the Res Uvae experimental farm, in Castell'Arquato, northern Italy. Isolates were grown on potato dextrose agar (PDA) and genomic DNA was extracted from fungal mycelium using the E.Z.N.A. Plant Miniprep kit (Omega Bio-tek) following manufacturer's instructions. DNA was stored at -20ºC. The primers EF1-688F [1] and EF1- 986R [2] were used to amplify and sequence part of the translation elongation factor 1-alpha (EF1-a). PCR products were purified with the High Pure PCR Product Purification kit (Roche Diagnostics) and sequenced by Macrogen Inc., Sequencing Center. Sequences were aligned with MEGA 7 [3], and manual adjustments made where necessary. GenBank sequences of closely related species of *Coniella* were selected based on their high similarity with the sequences generated here, using MEGA-BLAST (Sequences KX833626.1, KX833629.1, KX833606.1 and KX833622.1). Neighbour-joining phylogenetic analyses were performed with MEGA 7.

References

1. Alves, A.; Crous, P.W.; Correia, A.; Phillips, A.J.L. Morphological and molecular data reveal cryptic speciation in *Lasiodiplodia theobromae*. *Fungal Divers*. **2008**, 28, 1–13.
2. Carbone, I.; Kohn, L.M. A method for designing primer sets for speciation studies in filamentous ascomycetes. *Mycologia* **1999**, 91, 553–556.
3. Kumar, S.; Stecher, G.; Tamura, K. MEGA7: Molecular Evolutionary Genetics Analysis Version 7.0 for Bigger Datasets. *Mol. Biol. Evol*. **2016**, 33, 1870–1874.
